# Multiple uprising invasions of *Pelophylax* water frogs, potentially inducing a new hybridogenetic complex

**DOI:** 10.1038/s41598-017-06655-5

**Published:** 2017-07-26

**Authors:** Christophe Dufresnes, Mathieu Denoël, Lionel di Santo, Sylvain Dubey

**Affiliations:** 1 Department of Ecology & Evolution, Biophore Building, University of Lausanne, 1015 Lausanne, Switzerland; 2 Department of Animal & Plant Sciences, University of Sheffield, Western Bank, Sheffield, S3 7HF United Kingdom; 3 Laboratory of Fish & Amphibian Ethology, Behavioural Biology Unit, Freshwater and Oceanic Science Unit of Research (FOCUS), University of Liège, Quai van Beneden 22, 4020 Liège, Belgium; 4 Department of Biological Sciences, North Dakota State University, Fargo, ND USA; 5 Hintermann & Weber SA, Rue de l’Eglise-Catholique 9b, 1820 Montreux, Switzerland

## Abstract

The genetic era has revolutionized our perception of biological invasions. Yet, it is usually too late to understand their genesis for efficient management. Here, we take the rare opportunity to reconstruct the scenario of an uprising invasion of the famous water frogs (*Pelophylax*) in southern France, through a fine-scale genetic survey. We identified three different taxa over less than 200 km^2^: the autochthonous *P. perez*i, along with the alien *P. ridibundus* and *P. kurtmuelleri*, which have suddenly become invasive. As a consequence, the latter hybridizes and may now form a novel hybridogenetic complex with *P. perezi*, which could actively promote its replacement. This exceptional situation makes a textbook application of genetics to early-detect, monitor and understand the onset of biological invasions before they pose a continental-wide threat. It further emphasizes the alarming rate of amphibian translocations, both at global and local scales, as well as the outstanding invasive potential of *Pelophylax* aliens.

## Introduction

Species introductions and invasions represent a major threat to biodiversity worldwide. Amphibians are prominent examples of alien invaders, with multiple adverse effects on local fauna such as predation, ecological competition, spreading of diseases and genetic pollution of local relative species (reviewed by^1–3^). The efficient management of invasive amphibians primarily requires their early detection, proper identification and the long-term monitoring of their expansions. However, these steps can be challenging if introduced species are undistinguishable from related autochthonous taxa, with which they even often hybridize (e.g. refs 4–8). A reliable solution to trace biological invasions is the use of genetic tools^9, 10^. In fact, numerous cases of cryptic amphibian introductions, leading to sustainable alien populations (e.g. refs 5 and 11), or even massive hidden invasions (e.g. refs 8 and 12) have been brought to light thanks to recent molecular analyses. Unfortunately, most cases are noticed only after invaders have widely spread, resulting in complex situations difficult to contain, or irreversible (e.g. refs 4, 8 and 13), and not any more informative regarding the genesis of invasions.

These important issues are well-illustrated by European water frogs (*Pelophylax* sp.). This group involves multiple taxa distributed throughout the Western Palearctic^14^, and are characterized by a special mode of reproduction: hybridogenesis (reviewed in refs 15 and 16). This system involves the production of fertile hybrids (“kleptons”, abbreviated *kl* in nomenclature), that reproduce hemiclonally by eliminating one of the two parental genomes from the germline^17, 18^. For instance, hybridogenesis in *P*. kl. *esculentus* (of *P. lessonae* × *P. ridibundus* hybrid origin) triggers the elimination of the *P. lessonae* germline, and only the *P. ridibundus* genome is transmitted to their offspring^19^. Kleptons therefore act as genetic parasites^20^.

Water frogs are notorious invaders: many taxa have been introduced outward their natural ranges (*P. ridibundus* ^21^; *P. kurtmuelleri* ^11, 22, 23^; *P. bedriagae* ^13, 23, 24^; *P. bergeri* ^12^; *P. perezi* ^24^; and *P. shqipericus* ^25^). Some are currently expanding (*P. kurtmuelleri* in Italy) and are already present on a continental scale (*P. ridibundus* and *P. bergeri*), with catastrophic effects on local fauna such as predation, competition, as well as genetic introgression and/or hybridogenetic replacement of native water frogs^19, 26, 27^. Importantly, the ability of genome exclusion seems to vary geographically, depending on the hybridogenetic system from which the invaders is derived^16^, thus prompting for knowledge regarding the nature and origins of *Pelophylax* introductions. A major management issue is that morphological determination of these species is highly challenging^28–30^, and becomes even more confusing with the cryptic occurrence of exotic lineages throughout Western Europe.

One of the few Western European regions that has remained nearly free of water frogs is the Larzac plateau in southern France. Surveys from the 1970s did not mention water frogs in this region^31^ and more recent monitoring reported only scarce records at the early 2000s (MD, pers. obs.), despite widespread suitable breeding sites and close proximity from known lowland populations (refs 21, 32 ~15 km southeast and east of Larzac). Interestingly, the situation radically changed in the recent years: water frogs are currently massively expanding and have colonized most of the southern Larzac (MD, pers. obs.).

This situation provides a unique opportunity to characterize a new invasion of *Pelophylax* frogs *in statu nascendi*. To this end, we conducted a multilocus genetic survey in order to (1) determine the nature of expanding populations, (2) reconstruct the scenario of invasion, (3) get insights into the hybridization potential of the lineages involved and (4) assess the utility of morphological determination methods. We show that our study area is being invaded by three lineages simultaneously (undistinguishable by morphology), two of them potentially forming a new hybridogenetic complex. This exceptional pattern provides a textbook application of genetic methods for understanding the onset of biological invasions and guide their management, and further emphasizes the alarming rate and invasive potential of *Pelophylax* aliens.

## Results

### Molecular polymorphism

Our data combined mitochondrial *cyt-b* sequences and nuclear microsatellite genotypes (see Methods). We could include a total of 84 mitochondrial *cyt-b* haplotypes from the study area and other European *Pelophylax*, over 975 bp aligned (309 polymorphic sites, 261 of which are parsimony-informative). Microsatellite diversity ranged from 5 to 21 alleles (average: 12.5). Null alleles were detected for some loci/populations (Supplementary Table 1) and recoded as missing data. Locus *Rica5* featured a strong proportion of null alleles (>0.8 in some populations) and was therefore not included in population-based analyses (which relies on estimation of heterozygosity).

### MtDNA analyses

**Figure 1:**
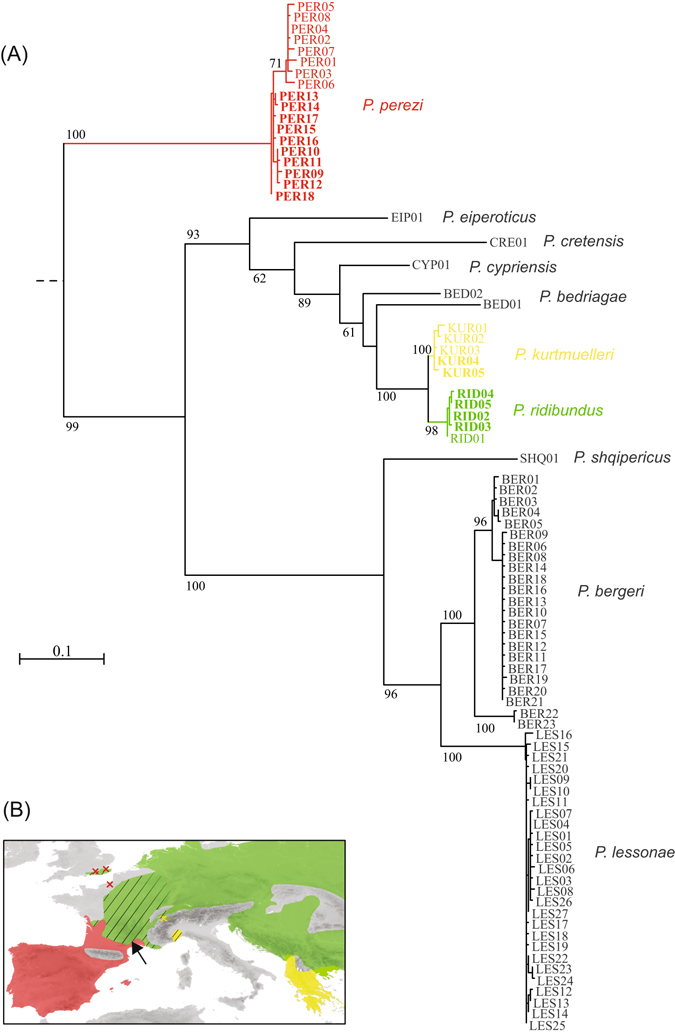
(**A**) Mitochondrial phylogeny of available *Pelophylax cytochrome-b* haplotypes and (**B**) distribution ranges of the three lineages sampled in the study area. Haplotypes sampled in the study area are highlighted in bold on the phylogeny. On the map, crosses and dashed areas represent introduced/invading populations; the arrow points to the location of the study area in southern France. The map was built using ArcGIS 9.3 (http://www.esri.com/arcgis/about-arcgis).

**Figure 2:**
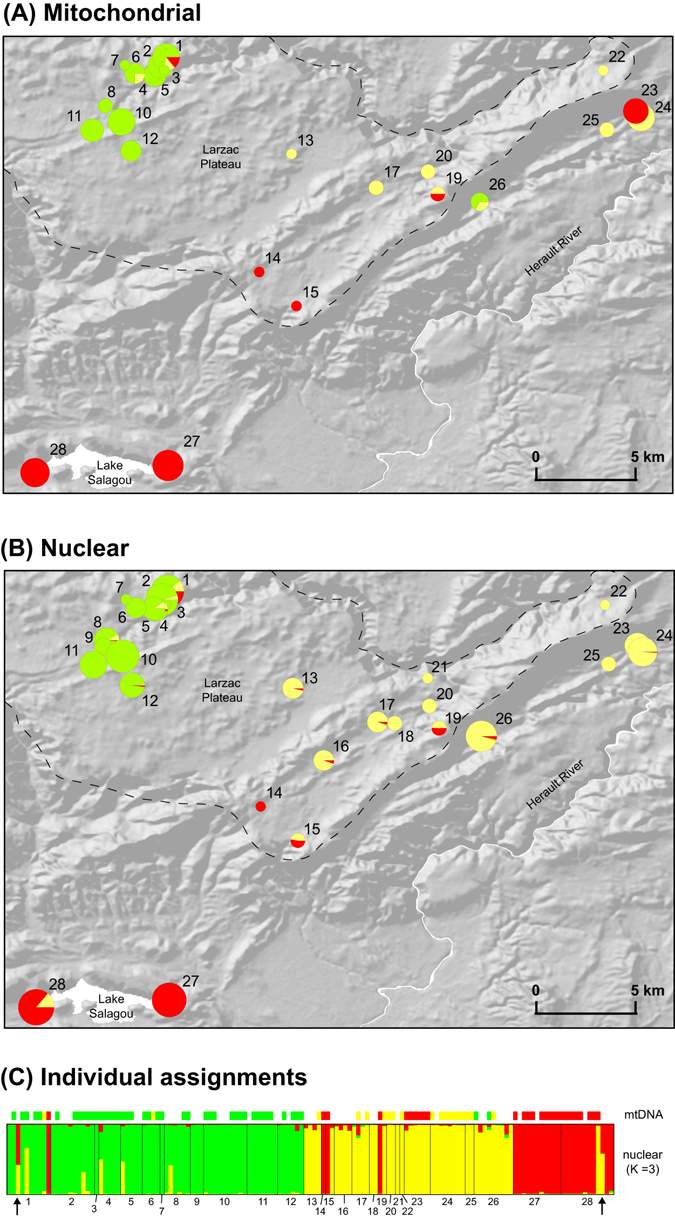
(**A**) Distribution of mitochondrial lineages, (**B**) nuclear clusters in the study area based on STRUCTURE average admixture proportions (for K = 3) and (**C**) individual STRUCTURE admixture proportions and mitotypes. Pie charts are proportional to sample sizes. Barplots show individual assignment for K = 3 (best solution according to the Pr(X│K) statistic) and mitotypes. The dash line delimits the Larzac Plateau. Red: *P. perezi*, yellow: *P. kurtmuelleri*, green: *P. ridibundus*. Arrows highlight the two individuals identified as a hypothetical *P. kurtmuelleri* × *P. perezi* new klepton. The maps were built using ArcGIS 9.3 (http://www.esri.com/arcgis/about-arcgis).

We identified three different mitochondrial lineages throughout the study area in southern France, belonging to three known *Pelophylax* monophyletic taxa (Fig. 1): the Perez’s frog (*P. perezi*), the marsh frog (*P. ridibundus*), and the Balkan frog (*P. kurtmuelleri*), with strong geographic association (Fig. 2A). Samples collected prior to the observed expansion (2006–2007) already featured all three mitotypes (*P. perezi* in loc. 14–15, 19; *P. ridibundus* in loc. 1, 7 and 10; *P. kurtmuelleri* in loc. 19–20). Analyses with restricted sequence length (514 bp), but enabling comparisons with published sequences of *P. ridibundus*, *P. kurtmuelleri* and *P*. cf. *bedriagae* from all over their ranges allowed to fully confirm the identification of Larzac’s haplotypes (Supplementary Figure 1).

MtDNA diversity in the study area was higher for the *P. perezi* lineage (10 haplotypes out of 27 samples; Supplementary Table 2) than the two others (*P. ridibundus*: 4 haplotypes out of 42 samples; *P. kurtmuelleri*: 2 haplotypes out of 19 samples; Supplementary Table 2).

### Nuclear DNA analyses

**Figure 3:**
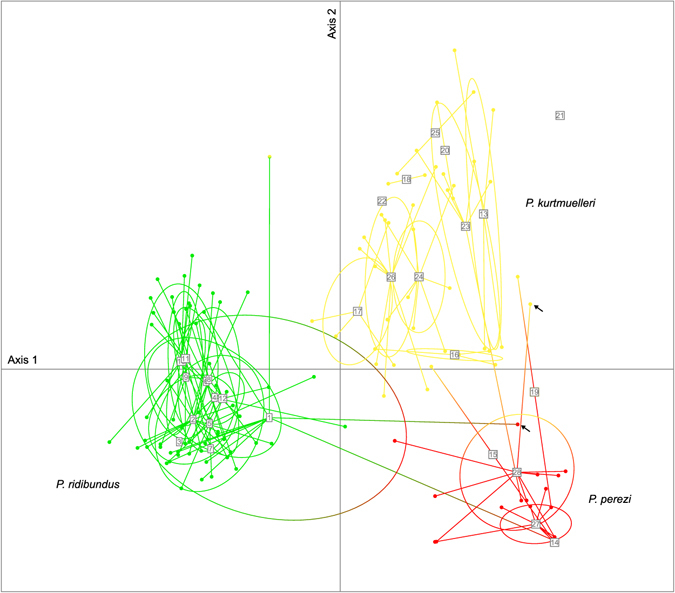
Principal Component Analysis (PCA) on microsatellite genotypes. Dots represent individuals, linked (lines) to populations (squared labels, with 80% inertia shown by ellipses). Colors represent the different clusters identified by STRUCTURE (K = 3) in the study area (cf. Fig. 2). Arrows highlight the two individuals identified *P. kurtmuelleri* × *P. perezi* hybrids from STRUCTURE, which may represent a new klepton.

The microsatellite data recovered a strong genetic structure corresponding to the three mtDNA lineages, as shown by the STRUCTURE analysis (when K = 3, Fig. 2B and C) and the PCA on individual genotypes (Fig. 3). Three STRUCTURE clusters (K = 3) is the solution recommended by the Pr(X│K) statistic, which only marginally increased for higher Ks (with stronger variance among and between runs, Supplementary Figure 2). Although substantial for K = 3, the ΔK statistic is the highest for K = 2, which groups *P. perezi* with *P. kurtmuelleri* along an otherwise similar picture (Supplementary Figure 2).

Although most individuals have matching nuclear and mitochondrial ancestries (Fig. 2C), we highlighted some interesting exceptions. First, several populations feature multiple cyto-nuclear discordances, i.e. nuclearly assigned to one cluster but carrying the mtDNA of another (e.g. loc. 23, 26, 28: *P. kurtmuelleri* individuals with *P. perezi* or *P. ridibundus* mtDNA). Second, some individuals had intermediate STRUCTURE admixture proportions consistent with hybridization (between *P. perezi* and *P. kurtmuelleri* in loc. 1 and 28, potentially representing a new hybridogenetic taxon, see Discussion; between *P. kurtmuelleri* and *P. ridibundus* in loc. 1–2, 4–5 and 8).

Population-based analyses suggested lesser nuclear differentiation between *P. ridibundus* and *P. kurtmuelleri* populations (average F_st  _ = 0.26) than compared to *P. perezi* (average F_st_ with *P. ridibundus* = 0.52; average F_st_ with *P. kurtmuelleri* = 0.42). Genetic structure within *P. ridibundus* populations was low (average F_st_ = 0.05), and more pronounced within *P. perezi* (F_st_ = 0.11) and *P. kurtmuelleri* (average F_st_ = 0.11) (Supplementary Table 3). In contrast with mtDNA, microsatellite heterozygosity was higher for *P. ridibundus* and *P. kurtmuelleri* than *P. perezi* (Supplementary Table 2). It was overall similar among the populations within species (*P. perezi*, H_o_ = 0.25–0.25; *P. ridibundus*, H_o_ = 0.49–0.59; *P. kurtmuelleri*: H_o_ = 0.57–0.68, Supplementary Table 1).

### Morphometric analyses

Seventy-four frogs from the study area were suitable for the morphometric analyses (17 *P. perezi*, 38 *P. ridibundus* and 19 *P. kurtmuelleri*; see Methods), in addition to the two *P. perezi* × *P. kurtmuelleri* hybrids. The PCA on morphometrics separated the three ratios relative to the length of the metatarsal tubercle (LMT, first axis) from the one relative to its height (HMT, second axis) (Supplementary Figure 3A). Overall, morphometrics of the three species strongly overlap on the PCA, although some *P. ridibundus* individuals slightly depart from the other species, especially *P. kurtmuelleri*. Accordingly, three out of four ratios significantly differ between *P. ridibundus* and *P. kurtmuelleri*, but none between *P. kurtmuelleri* and *P. perezi* (Tuckey’s HSD tests, Supplementary Figure 3B).

## Discussion

Our study allowed to accurately reconstruct an uprising invasion of the notorious *Pelophylax* water frogs over a fine-scale spatiotemporal framework, informing on several crucial aspects regarding the invasive amphibians’ issues. We show that this invasion involves multiple taxa, indicative of a myriad of independent introductions at narrow geographic and time scales. Importantly, it may induce a new hybridogenetic complex that, if not contained, could represent a serious threat to the native water frog species occurring in this part of Europe.

### Multiple *Pelophylax* invasions in southern France

Our study identified three distinct *Pelophylax* species in Larzac, an area nearly-free of water frogs a decade ago, as well as the nearby lowlands. These include the Balkan’s frog (*P. kurtmuelleri*), native from Greece and Albania; its sister species the Eurasian marsh frog (*P. ridibundus*), widely distributed throughout Eastern and Central Europe; and the Perez’s frog (*P. perezi*), which inhabits Spain, Portugal and southern France. Their presence is demonstrated by unequivocal mitochondrial evidence, further supported by reciprocal nuclear clustering of microsatellite genotypes. Importantly, whereas previous genetic analyses could not reliably discriminate between *P. ridibundus* and *P. kurtmuelleri* ^33, 34^, we show that the mitochondrial marker *cyt-b* is powerful enough to distinguish both taxa in monophyletic clades, even when accounting for their strong intraspecific diversity (Fig. 1, Supplementary Figure 1). However, none of the three species detected in Larzac could be discriminated by classic morphological criteria.

Being naturally distributed in eastern parts of Europe, *P. ridibundus* and *P. kurtmuelleri* are clearly exotic in southern France. These taxa are known European invaders, originating from uncontrolled releases, linked in some cases with the frog-leg industry (*P. ridibundus*). Given previous records that showed no water frogs over Larzac in the 1970s^31^ (although one of our locality, loc. 19, was not surveyed), both species were probably first introduced between ten and forty years ago in Larzac, and expanded throughout the plateau in recent years (this study). The current geographic pattern suggests that several independent introduction events contributed to the presence of *P. ridibundus* in the west and of *P. kurtmuelleri* in the east of the study area. Releases were probably numerous and of various origins: several haplotypes were sampled for each species, and different mitochondrial/nuclear lineages co-occur in both areas. This is exacerbated in the west (loc. 1–12), where multiple introduction events have been reported by locals (from Lake Salagou and potentially other areas, MD pers. com.), resulting in a mixture of different taxa.

The origin of the third Larzac’ water frog, *P. perezi*, is unclear. It may either involve illegal releases from presumably native southern populations (e.g. loc. 27–28) and/or natural expansion northwards. Yet, from our samples, the presence of *P. perezi* seems to also predate the recent demographic explosion, so its historical, cryptic persistence in Larzac cannot be excluded. In contrast with the two other expanding lineages, the high mtDNA diversity argues for a long-term demographic stability of *P. perezi* populations, and thus their legitimate occurrence in the area. Microsatellite gives a somehow conflicting signal, i.e. lower diversity of *P. perezi* compared to *P. ridibundus* and *P. kurtmuelleri*. However, this might be because our loci were originally developed from the *P. ridibundus* species complex: microsatellite diversity is known to severally drop when reused in congeneric species, proportionally to their genetic distance^35^. In any case, the contemporary situation of *P. perezi* in Larzac rather seems precarious compared to the other expanding species, which may actually replace it in the area (see below).

After years of scarce records, the sudden colonization of Larzac by water frogs is puzzling. These range expansions may testify that introduced lineages are more adapted to the dry conditions of Larzac (i.e. the Greek/Albanian *P. kurtmuelleri*), and/or be a response to climate change. Temperature, evapotranspiration and aridity indices rapidly increased in the last three decades over southern France, leading to a northward extension of the Mediterranean climate (by 1 to 2.5°N;^36^). As ectotherms, amphibians are highly sensitive to thermal and hydric environmental modifications^37^. Detailed ecological surveys combined with niche modelling analyses, would help testing the role of climate change in these water frog invasions, and especially understand whether the natural range of *P. perez*i extended northward. In parallel, the numerous suitable waterbodies in the area^38^ along with the strong dispersal capability of water frogs^39^ likely triggered the rapid colonization. This high connectivity is also illustrated by low genetic differentiation among the populations of each taxon. Hence, we predict that *P. ridibundus* (expanding southeastward) will soon meet *P. kurtmuelleri* (expanding westward) in the center of our study area (between loc. 12 and 13). These two species being known for their distinctive advertisement calls^28^, future monitoring of the invasions should include bioacoustic protocols, providing a cheap and reliable alternative to genetic tools in this case.

### Hybrization between Larzac water frogs

Our genetic survey provides valuable insights on the hybridizability and especially hybridogenetic potential of alien water frogs. This aspect is not-well documented in the *Pelophylax* literature and so far only three systems were identified (*P*. kl. *esculentus*, *P*. kl. *grafi*, *P*. kl. *hispanicus*;^16^). Hybridization mechanisms are crucial to consider in the context of biological invasions, as they may induce genetic pollution (through introgressive hybridization) or cause the elimination of parental germlines (through hybridogenesis), in both cases eventually leading to the complete replacement of local taxa (e.g refs 8, 19 and 20). Importantly, effects and potential for hybridogenesis were shown to vary geographically, depending on the system and origin of the invaders^16, 40^; it thus deserves special attention in new invasion cases.

We found evidence for hybridization with genetic introgression between the alien *P. ridibundus* and *P. kurtmuelleri*. Several individuals feature intermediate admixture proportions at nuclear markers (Fig. 2B), while other show symmetric cyto-nuclear discordance (*P. ridibundus* carrying *P. kurtmuelleri* mtDNA and vice-versa), indicative of backcrossing. This indicates that the two marsh frog species *P. ridibundus* and *P. kurtmuelleri* are able to successfully mate, which is not surprising given their young evolutionary history (~1.8 My^14^). In amphibians, reproductive isolation in the wild is usually reached at Pleistocene divergences, although this depends on radiations (3–5 My^41, 42^,) and the time since contact^43^. Introgressive hybridization was also reported between the invasive *P. ridibundus* and *P. bedriagae* in Belgium^29^, despite four million years of independent evolution^14^.

**Figure 4:**
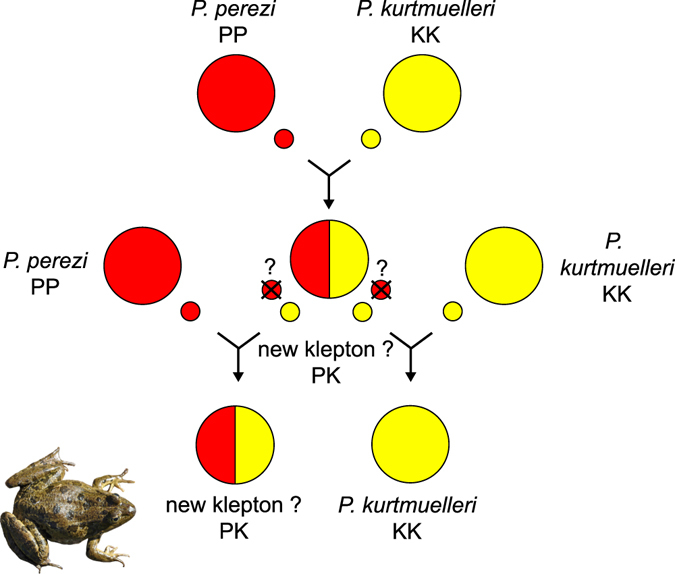
Hypothesis of a new *Pelophylax* hybridogenetic complex in southern France. Large circles illustrate genomes; small circles illustrate gametes. Crosses between *P. perezi* and *P. kurtmuelleri* would form a new klepton. As their *P. perezi* germline might be eliminated (as in the *P*. kl *grafi* complex), kleptons may only produce *P. kurtmuelleri* offspring (when mating with *P. kurtmuelleri*) or *P. kurtmuelleri* × *P. perezi* klepton offspring (when mating with *P. perezi*). If verified, these mechanisms would precipitate the decline of *P. perezi*. Photo of a suspected new klepton (loc. 1): Mathieu Denoël.

In parallel, two observations are in line with a potential novel hybridogenetic system between *P. perezi* and *P. kurtmuelleri*, which would then form a new klepton. First, two individuals were identified as *P. perezi* × *P. kurtmuelleri* hybrids with F1-like admixture proportions, consistent with this new klepton (loc. 1 and 28, Fig. 2B). Second, many *P. kurtmuelleri* individuals carried *P. perez*i’s mtDNA (loc. 23, 28). Such asymmetric cyto-nuclear discordance is a known echo of hybridogenetic dynamics^44^; in our case, it can only result from the backcrossing of female hybrids (from *P. perezi* mothers) by *P. kurtmuelleri* males, thus restoring two copies of the nuclear *P. kurtmuelleri* genome while maintaining the maternally-transmitted *P. perezi* mtDNA. The fact that we did not find the reverse pattern (*P. perezi* carrying *P. kurtmuelleri* mtDNA) is thus compatible with a hypothetical elimination of the *P. perez*i germline in hybrids, which would then only contribute gametes with the *P. kurtmuelleri* genome (Fig. 4). This hypothetical mechanism, comparable to the *P*. kl. *esculentus* and *P*. kl *grafi* hybridogenetic complexes, would promote the replacement of *P. perezi* by *P. kurtmuelleri*, thus accounting for its striking invasion in southern France.

*Pelophylax ridibundus*, the sister species of *P. kurtmuelleri*, is known to hybridize with *P. perezi* to form the Graf’s frog (*P*. kl*. grafi*). It is thus legitimate to assume that *P. kurtmuelleri* could have the potential to form a similar hybridogenetic complex with *P. perezi* (Fig. 4), which would also not be unexpected given the old origin of hybridogenesis in water frogs^15^. However, it contradicts previous studies which suggested that southeastern European marsh frogs do not reproduce in this mode^16^, which may then depend on the partner species involved. Further research is obviously needed to characterize this potential new system, notably to determine genome exclusion in the germinal cells of hybrids, and the effects on *P. perezi* populations. Given the possible interactions with a third hybridogenetic species, *P. ridibundus*, this system makes a remarkable natural laboratory for the understanding of these mechanisms. Especially, the opportunity for crosses between different types of potentially sympatric kleptons (*P*. kl. *grafi*, hypothetical *P. kurtmuelleri* × *P. perezi* kleptons, as well as kleptons featuring admixed *P. ridibundus* × *P*. *kurtmuelleri* genomes, see above) might provide novel insights regarding their occurrence and evolution.

It is surprising however, that we failed to detect *P*. kl *grafi*, i.e. the klepton between *P. perezi* and *P. ridibundus*, among our samples; this taxon usually coexists with *P. perezi* in southern France^21^. We see several potential reasons for this absence/misdetection. First, we included only one area where the natural occurrence of *P*. kl *grafi* was confirmed, i.e. Lake Salagou (loc. 27–28). Micro-differences in breeding periods and habitat choice (resulting from habitat partitioning, as shown in that *P*. kl *esculentus* complex^45^ and between *P. perezi* and *P*. kl *grafi* ^21^), along with yearly fluctuations in relative frequencies may all have contributed to an involuntary bias towards *P. perezi* in our sampling of Salagou. Second, in areas where marsh frogs have become invasive, it is not excluded that *P*. kl *grafi* has been replaced ecologically or genetically, in the same way as *P. perezi*. Cyto-nuclear discordance in *P. kurtmuelleri* individuals (carrying *P. ridibundus* mtDNA) could in fact stem from past hybridization with *P*. kl *grafi*.

### Conservation applications

Our results have direct implications for the management of *Pelophylax* invaders. First, we demonstrate the necessity of molecular tools to detect these invasions. No morphometric criteria allowed to reliably discriminate between the *Pelophylax* species identified in southern France, as shown for the *P*. kl. *esculentus* complex^29, 46^. The significant morphological variations we reported between *P. ridibundus* and other lineages might be linked to heterogeneous environmental conditions rather than actual interspecific differences, given that this taxon is currently distributed over a separated part of the study area. This species is anyway known to be highly variable phenotypically^47^.

Second, our study emphasizes the need for early detection of these invasions. Here only a few years were sufficient for *Pelophylax* aliens to conquer large areas, even in arid environments. Amphibian invaders are often detected too late, and in the meanwhile had already established wide invasive ranges, impeding their efficient eradication and compromising native species (e.g. refs 12 and 23). The development of environmental DNA monitoring programs may contribute to the systematic early-detection of invaders in the future, providing a sufficiently exhaustive barcoding database^48^.

Third, we raise the flag on extremely frequent human translocations, both at the global and the regional scale, a central cause of the biological invasion problem^1, 2, 49^. Here alien lineages were found in every parts of the study area, co-existing in several localities, and even in supposedly non-affected neighboring populations (loc. 28). In addition to involuntary releases linked to international trade, amphibians are increasingly introduced for ornamental and culinary purposes in private ponds, without concern regarding their taxonomic nature and the ecological consequences. We recommend immediate conservation measures to limit such translocations, notably through a more stringent legislative policy on the matter, along with actions to raise awareness to the general public.

Fourth, this situation demonstrates the outstanding hybridizing potential of *Pelophylax*. Here, it could form a new, artificial, hybridogenetic complex between the Balkan’s and Perez’s frogs, eventually driving the local extinction of the latter. While this issue deserves further investigations, it can be paralleled with other hybridogenetic systems formed in invasive contexts (e.g. *P*. kl *esculentus* in Western Europe, *P*. kl *hispanicus*-like in Switzerland, *P*. kl *grafi* in southern France). Importantly, it alarms on the risk posed by *P. kurtmuelleri* on native green frogs throughout Europe (here, *P. perezi*).

In respect to these points, we recommend the eradication of identified alien populations. Immediate actions should be taken to prevent the ongoing colonization of *Pelophylax* to extend outside the plateau. Such measure seems conceivable due to the remote geographic situation of Larzac. However, it might be more complicated in the well-connected lowlands, where amphibian communities are already a mixture of native and exotic species^32^. The management of hybridizing, morphologically similar species like water frogs are particularly problematic in areas of long-term occurrence, since native populations may have been secretly replaced by invasive taxa without noticeable demographic changes.

## Methods

### Sample collection

A total of 139 water frogs from 28 localities were captured with dip-nets during several breeding seasons (spring-summer 2006–2015) throughout the study area, which encompasses the Larzac Plateau (loc. 1–22), the valley of the Hérault River in the southeast (loc. 23–26) and Lake Salagou in the southwest (loc. 27–28). In addition, we further included samples from the natural ranges of *P. kurtmuelleri* (n = 5, from Albania) and *P. perezi* (n = 9 from different Spanish localities) to be used as reference in phylogenetic analyses, complementing published data (see below). Supplementary Table 1 provides detailed sampling information.

Most individuals were measured with a digital caliper and a ruler for snout-ventral length (SVL), tibia length (LTi), toe length (LTo), and for the metatarsal tubercle length (LMT) and height (HMT). Tissues samples for DNA analyses were obtained by toe clipping, and preserved in 96% ethanol at 4 °C. Procedures were approved by the local and national ethics committees for animal experiments (DREAL Languedoc-Roussillon and Conseil National de la Conservation de la Nature) and performed in accordance with their guidelines and regulations.

### Molecular labwork

DNA was extracted using the Qiagen Biospring robotic workstation. We sequenced the mitochondrial *cytochrome-b* (975 bp) in 102 individuals (n = 88 from the study area and 14 from natural ranges), using published primers (cytb_60 F and cytb_60 R from^50^) and methods^12^.

We genotyped all individuals from the study area for eight polymorphic microsatellites in water frogs (*Rica5*, *Rica1b5*, *Rica1b6*, *Re1Caga10*, *ReGa1a23, Rrid013A*, *Rrid059A*, and *Res16*). These loci have the power to distinguish species from the *P*. kl. *esculentus* complex as well as to discriminate other exotic taxa^29^. These were amplified in two multiplex PCRs, diluted, and ran to an ABI3130 Genetic Analyzer, strictly following the protocols described in ref. 12. We discarded a ninth marker (*Rica18*) used in ref. 12 from subsequent analyses, which was not amplifying in the Larzac’s water frogs.

### Genetic analyses

We combined our new sequences (n = 102) with published ones to build a maximum-likelihood phylogenetic tree of mitochondrial *cyt-b* haplotypes with PhyML^51^. We used a GTR + I + G model of sequence evolution (MrAIC^52^,) and tested branch support by 1 000 bootstrap replicates. Published sequences correspond to all European *Pelophylax* spp. *cyt-b* haplotypes available on GenBank with a similar coverage (975 bp), and include *P. bedriagae*, *P. bergeri*, *P. cretensis*, *P. cypriensis*, *P. epeiroticus*, *P. kurtmuelleri*, *P. lessonae*, *P. perezi*, *P. ridibundus* and *P. shqipericus* (see Supplementary Table 1 for details). *Pelophylax nigromaculatus* was used as outgroup. Mitochondrial diversity was assessed by computing haplotype (H_d_) and nucleotide diversity (п) in DNASP^53^. Moreover, to ascertain species identification, especially between closely-related marsh frogs (see Results), we analyzed a shorter portion of *cyt-b* (514 bp) to include published sequences from previous phylogeographic work^14^ that exhaustively covers the intraspecific diversity of these species.

Microsatellite genotypes were checked and corrected for null alleles using Micro-Checker^54^. We then performed several analyses of genetic structure. First, we used STRUCTURE^55^ to cluster individuals into K groups, testing from K = 1 to 11. We computed ten replicate runs per K, each consisting of 100’000 iterations after 10’000 of burnin. The numbers of groups best-explaining the data were retained considering the average log-likelihood Pr(X│K) and the ΔK statistics^55, 56^, computed in STRUCTURE HARVESTER^57^. Their replicate runs were combined with CLUMPP^58^ and graphically represented using DISTRUCT^59^. Second, we performed a Principal Component Analysis (PCA) on individual genotypes using the R packages *adegenet* and *ade4*. Third, we computed pairwise genetic distances (F_st_) between populations with sufficient sample sites (n > 6) in Fstat^60^, pooling some proximate localities (distant by <500 m; loc. 4–5, 8–9 and 17–18). We also estimated observed heterozygosity (H_o_) for these populations, as well as for the identified taxa (see Results), considering only individuals confidently assigned to the corresponding nuclear cluster (STRUCTURE admixture proportion above 0.95). One marker (*Rica5*) was not considered in population-based analyses due to the high frequency of null alleles (see Results).

### Morphometric analyses

To test whether the identified taxa can be distinguished by morphometrics, we selected individuals with available measurements data that were confidently assigned to the corresponding nuclear cluster (see the end of last section), and at least 40 mm long (SVL). We also included the two individuals identified as *P. perezi* × *P. kurtmuelleri* hybrids, which might represent a new hybridogenetic taxon (see Results and Discussion). We considered four ratios of measurements usually tested as discriminants between sympatric species of *Pelophylax* frogs^30, 46^: toe length/metatarsal tubercle length (Lto/LMT), tibia length/metatarsal tubercle length (Lti/LMT), tibia length/metatarsal tubercle height (LTi/HMT), and snout-ventral length/metatarsal tubercle length (SVL/LMT).

We performed a PCA on these four variables (log-transformed) for the selected individuals using the R package *ade4*. In addition, we tested whether they significantly differ between taxa with Tuckey’s HSD tests.

## Electronic supplementary material

Supplementary Information

## References

[1] Kraus, F. *Alien reptiles and amphibians: A scientific compendium and analysis*. Dordrecht: Springer (2009).

[2] Kraus F (2015). Impact from invasive reptiles and amphibians. Annu. Rev. Ecol. Evol. Syst..

[3] Bucciarelli GM, Blaustein AR, Garcia TS, Kats LB (2014). Invasion complexities: the diverse impacts of nonnative species on amphibians. Copeia.

[4] Johnson JR, Fitzpatrick BM, Shaffer HB (2010). Retention of low-fitness genotypes over six decades of admixture between native and introduced tiger salamanders. BMC Evol. Biol..

[5] Meilink WRM, Arntzen JW, van Delft JJCW, Wielstra B (2015). Genetic pollution of a threatened native crested newt species through hybridization with an invasive congener in the Netherlands. Biol. Conserv..

[6] Shaffer HB (2015). Conservation genetics and genomics of amphibians and reptiles. Annu. Rev. Anim. Biosci..

[7] Dufresnes C, Dubey S, Ghali K, Canestrelli D, Perrin N (2015). Introgressive hybridization of threatened European tree frogs (*Hyla arborea*) by introduced *H. intermedia* in Western Switzerland. Conserv. Genet..

[8] Dufresnes C (2016). Massive genetic introgression in threatened northern crested newts (*Triturus cristatus*) by an invasive congener (*T. carnifex*) in Western Switzerland. Conserv. Genet..

[9] Lawson Handley LJ (2011). Ecological genetics of invasive alien species. BioControl.

[10] Cristescu ME (2015). Genetic reconstructions of invasion history. Mol. Ecol..

[11] Ficetola, G. F. & Scali, S. *Invasive amphibians and reptiles in Italy*. Atti, VIII Congresso Nazionale Societas Herpetologica Italica 335–340 (2010).

[12] Dufresnes, C. *et al*. Cryptic invasion of Italian pool frogs (*Pelophylax bergeri*) across Western Europe unraveled by multilocus phylogeography. *Biol. Invasions* In Press. doi:10.1007/s10530-016-1359-z (2017).

[13] Holsbeek G (2008). A cryptic invasion within an invasion and widespread introgression in the European water frog complex: consequences of uncontrolled commercial trade and weak international legislation. Mol. Ecol..

[14] Lymberakis P (2008). Mitochondrial phylogeography of *Rana* (*Pelophylax*) populations in the Eastern Mediterranean region. Mol. Phylogenet. Evol..

[15] Ogielska, M. Development and reproduction of amphibian species, hybrids, and polyploids. In: Ogielska M, editor. *Reproduction of amphibians*. Enfield: Science Publishers (2009).

[16] Holsbeek G, Jooris R (2010). Potential impact of genome exclusion by alien species in the hybridogenetic water frogs (*Pelophylax esculentus* complex). Biol. Invasions.

[17] Schultz RJ (1969). Hybridization, unisexuality, and polyploidy in the teleost *Poeciliopsis* (Poeciliidae) and other vertebrates. Am. Nat..

[18] Tunner HG (1974). Die klonale Struktur einer Wasserfroschpopulation. J. Zool. Syst. Evol. Res..

[19] Vorburger C, Reyer HU (2003). A genetic mechanism of species replacement in European waterfrogs?. Conserv. Genet..

[20] Leuenberger J, Gander A, Schmidt BR, Perrin N (2014). Are invasive marsh frogs (*Pelophylax ridibundus*) replacing the native *P. lessonae*/*P. esculentus* hybridogenetic complex in Western Europe? Genetic evidence from a field study. Conserv. Genet..

[21] Pagano A, Crochet PA, Graf JD, Joly P, Lodé T (2001). Distribution and habitat use of water frog hybrid complexes in France. Global. Ecol. Biogeogr..

[22] Jørgensen K (1999). Latterfrøer i Fælledparken!. Nord Herpet Foren..

[23] Dubey S, Leuenberger J, Perrin N (2014). Multiple origins of invasive and ‘native’ water frogs (*Pelophylax* spp.) in Switzerland. Biol. J. Linn. Soc..

[24] Holsbeek G, Mergeay J, Volckaert FAM, De Meester L (2010). Genetic detection of multiple exotic water frog species in Belgium illustrates the need for monitoring and immediate action. Biol. Invasions.

[25] Domeneghetti D, Bruni G, Fasola M, Bellati A (2013). Discovery of alien water frogs (gen. *Pelophylax*) in Umbria, with first report of *P. shqipericus* for Italy. Acta Herpetol..

[26] Schmeller DS, Pagano A, Plénet S, Veith M (2007). Introducing water frogs - Is there a risk for indigenous species in France?. C. R. Biol..

[27] Paunović A, Bjelić-Čabrilo O, Šimić S (2010). The diet of water frogs (*Pelophylax esculentus* “complex”) from the Petrovaradinski Rit marsh (Serbia). Arch. Biol. Sci..

[28] Nöllert, A., & Nöllert, C. *Guide des amphibiens d’Europe*. Paris: Delachaux et Niestlé (2003).

[29] Holsbeek G, Maes GE, De Meester L, Volckaert FAM (2009). Conservation of the introgressed European water frog complex using molecular tools. Mol. Ecol..

[30] Plötner J (2010). Applications and limits of morphological methods for species determination of European water frogs (*Pelophylax esculentus* complex). Z. Feldherpetol..

[31] Gabrion, J. *La néoténie chez Triturus helveticus Raz. Etude morphofonctionnelle de la fonction thyroïdienne*. PhD Thesis, Université des Sciences et Techniques du Languedoc, Montpellier (1976).

[32] Geniez, P. & Cheylan, M. *Les amphibiens et les reptiles du Languedoc-Roussillon et régions limitrophes. Atlas biogéographique*. Mèze and Paris: Biotope and Museum National d’Histoire Naturelle (2012).

[33] Akin C (2010). Phylogeographic patterns of genetic diversity in eastern Mediterranean water frogs were determined by geological processes and climate change in the Late Cenozoic. J. Biogeogr..

[34] Plötner, J. *et al*. Genetic divergence and evolution of reproductive isolation in Eastern Mediterranean water frogs. In: Evolution in Action (ed. Glaubrecht, M.) 373-403 (Springer Verlag 2010).

[35] Dufresnes C, Brelsford A, Béziers P, Perrin N (2014). Stronger transferability but lower variability in transcriptomic- than in anonymous microsatellites: evidence from Hylid frogs. Mol. Ecol. Resour..

[36] Lelièvre, F., Sala, S., Ruget, F. & Volaire, F. *Evolution climatique du Sud de la France 1950-2009, Projet CLIMFOUREL PSDR‐3, Régions L‐R, M‐P, R‐A. Série Les Focus PSDR3*. Available at: http://www.opcc-ctp.org/etudes/FOCUS-PSDR3-CLIMFOUREL_Clim_Chgt.pdf (2011).

[37] Blaustein AR (2010). Direct and indirect effects of climate change on amphibian populations. Diversity.

[38] Denoël M, Ficetola GF (2015). Using kernels and ecological niche modeling to delineate conservation areas in an endangered patch-breeding phenotype. Ecol. Appl..

[39] Tunner, H. G. Locomotory behaviour in water frogs from Neusiedlersee (Austria, Hungary). 15 km migration of *Rana lessonae* and its hybridogenetic associate *Rana esculenta*. In: Korsos V and Kiss I, editors. Proccedings of the Sixth Ordinary General Meeting Societas Europaea Herpetologica. Budapest (1992).

[40] Hotz H (1985). *Rana ridibunda* varies geographically in inducing clonal gametogenesis in interspecies hybrids. J. Exp. Zool..

[41] Dufresnes C (2014). Inferring the degree of incipient speciation in secondary contact zones of closely related lineages of Palearctic green toads (*Bufo viridis* subgroup). Heredity.

[42] Dufresnes C (2015). Timeframe of speciation inferred from secondary contact zones in the European tree frog radiation (*Hyla arborea* group). BMC Evol. Biol..

[43] Dufresnes C (2016). Empirical evidence for large X-effects in animals with undifferentiated sex chromosomes. Sci. Reports.

[44] Plötner J (2008). Widespread unidirectional transfer of mitochondrial DNA: a case in western Palearctic water frogs. J. Evol Biol..

[45] Pagano A, Joly P, Plénet S, Lehman A, Grolet O (2001). Breeding habitat partitioning in the *Rana esculenta* complex: The intermediate niche hypothesis supported. Ecoscience.

[46] Pagano A, Joly P (1999). Limits of the morphometric method for field identification of water frogs. Alytes.

[47] Pagano A, Joly P, Hotz H (1997). Taxon composition and genetic variation of water frogs in the mid-Rhône floodplain. C. R. Acad. Sci. III.

[48] Dejean T (2012). Improved detection of an alien invasive species through environmental DNA barcoding: The example of the American bullfrog *Lithobates catesbeianus*. J. Appl. Ecol..

[49] Smith, R. K., & Sutherland, W. J. *Amphibian conservation: global evidence for the effects of interventions*. Exeter: Pelagic Publishing Ltd. (2014).

[50] Hofman S, Pabijan M, Dziewulska-Szwajkowska D, Szymura JM (2012). Mitochondrial genome organization and divergence in hybridizing central European waterfrogs of the *Pelophylax esculentus* complex (Anura, Ranidae). Gene.

[51] Guidon S, Gascuel O (2003). A simple, fast, and accurate algorithm to estimate large phylogenies by maximum likelihood. Syst, Biol..

[52] Nylander, J. A. A. *MrAIC.pl. Program distributed by the author*. Evolutionary Biology Centre, Uppsala University. Available at: https://github.com/nylander (2004).

[53] Librado P, Rozas J (2009). DNASP v5: a software for comprehensive analysis of DNA polymorphism data. Bioinformatics.

[54] van Oosterhout C, Hutchinson WF, Wills DPM, Shipley P (2004). MICRO-CHECKER: software for identifying and correcting genotyping errors in microsatellite data. Mol. Ecol. Resour..

[55] Pritchard JK, Stephens M, Donnelly P (2000). Inference of population structure using multilocus genotype data. Genetics.

[56] Evanno G, Regnaut S, Goudet J (2005). Detecting the number of clusters of individuals using the software STRUCTURE: a simulation study. Mol. Ecol..

[57] Earl DA, VonHoldt BM (2012). STRUCTURE HARVESTER: a website and program for visualizing STRUCTURE output and implementing the Evanno method. Conserv. Genet. Resour..

[58] Jakobsson M, Rosenberg NA (2007). CLUMPP: a cluster matching and permutation program for dealing with label switching and multimodality analysis of population structure. Bioinformatics.

[59] Rosenberg NA (2004). DISTRUCT: a program for the graphical display of population structure. Mol. Ecol. Notes.

[60] Goudet J (1995). FSTAT (version 1.2): a computer program to calculate F-Statistics. J. Hered..

