# Supplementary material for: Multiple uprising invasions of Pelophylax water frogs, potentially inducing a new hybridogenetic complex
Source: Sci Rep. 2017 Jul 26;7:6506. doi: 10.1038/s41598-017-06655-5 (PMC5529583; doi:10.1038/s41598-017-06655-5)

Multiple uprising invasions of *Pelophylax* water frogs, potentially inducing a new hybridogenetic complex

Christophe Dufresnes, Mathieu Denoël, Lionel di Santo and Sylvain Dubey

Supplementary Information

Supplementary Table 1: Locality information

Supplementary Table 2: Diversity of each taxon

Supplementary Table 3: Pairwise genetic distances

Supplementary Figure 1: Phylogenetic analyses of a portion of *cyt-b* (514bp aligned)

Supplementary Figure 2: STRUCTURE statistics

Supplementary Figure 3: Morphometric analyses

Supplementary Table 1A: Locality information. n indicates samples sizes used in morphometric (morpho), mitochondrial (mtDNA) and nuclear (µsat) analyses. Lat.: latitude; Long.: longitude. H_o: observed heterozygosity for populations with sufficient sample sizes ($n \geq 6$), some of which has been pooled (4-5, 8-9, 17-18). Null: detection of null alleles (genotypes were subsequently corrected).

ID	Locality	Lat.	Long.	N_{morpho}	N_{mtDNA}	$N_{\mu sat}$	H_o	Null
1	Ville Vieille	43.89888	3.40549	6	7	10	0.58	*Rica1b5*, *Rica5*
2	Four Banal	43.89393	3.40214	7	6	10	0.55	*Rica5*
3	Château de Sorbs SW	43.89109	3.40428	0	1	1	-	-
4	Baume Vieille W	43.88821	3.39876	4	5	5	0.57	*Rica1b6*
5	Moulin W	43.88874	3.39742	2	3	5	-	*Res16*
6	Sotch de Caylus	43.88920	3.38701	4	4	4	-	-
7	Corombelle	43.89413	3.38113	0	1	1	-	-
8	Cros Farm	43.86995	3.36963	3	2	6	0.56	-
9	Cros réservoir	43.86604	3.36769	0	0	3	-	-
10	Bergerie de l'Hôpital	43.86057	3.37910	8	7	10	0.48	*Res16*, *ReGa1a23*
11	Bagnelades	43.85557	3.36178	5	5	7	0.49	-
12	St-Michel Le Laquet	43.84344	3.38469	0	4	6	0.58	-
13	Besses	43.84160	3.47962	0	1	4	-	-
14	Source du Goutal (Trop plein)	43.77180	3.46051	0	1	1	-	-
15	Devois la Trivalle	43.75166	3.48255	0	1	2	-	-
16	Ferrussac	43.79902	3.49786	0	0	4	-	-
17	Coulet Tournant	43.82167	3.52965	0	2	4	0.59	-
18	Coulet NE	43.82089	3.53980	2	0	2	-	-
19	Mare du Goutal	43.81801	3.56600	0	2	2	-	-
20	Natges	43.83119	3.56029	0	2	2	-	-
21	Rancas N	43.84761	3.55923	0	0	1	-	-
22	Bairades	43.89098	3.66388	0	1	1	-	-
23	Lac de Condamine	43.86694	3.68313	5	6	6	0.57	*Res16*
24	Clapas de Lamathe	43.86312	3.68620	8	7	8	0.68	*Rica5*, *Res16*
25	Source de la Pradasse	43.85593	3.66607	1	2	2	-	-
26	Source de la Buèges	43.81332	3.59095	2	3	9	0.58	*Rica5*, *Res16*, *ReGa1a23*
27	Penchant du Salagou	43.65746	3.40653	7	9	11	0.25	*Rica5*
28	Octon (Plage - Salagou)	43.65320	3.32799	10	8	12	0.25	*Rica1b5*, *Rica1b6*, *Rica5*

Supplementary Table S1B: Samples used as references in phylogenetic analyses of *cyt-b* (975bp aligned).

GenBank	Species	Origin	Reference
KP260932	*P. bedriagae*	Jordan	-
-	*P. bedriagae*	Switzerland, Luzern	Dufresnes et al. 2017, Invasion Biol..
-	*P. bergeri*	Italy, Switzerland, France	Dufresnes et al. 2017, Invasion Biol..
KM677928	*P. cretensis*	Greece, Crete	Hofman et al. 2016, Mitochondrial DNA 27: 1995-1996
KP814009	*P. cypriensis*	Greece, Cyprus	Hofman et al. 2016, Mitochondrial DNA 27: 3434-3437
KP814010	*P. epeiroticus*	Greece	Hofman et al. 2016, Mitochondrial DNA 27: 3434-3437
KP814011	*P. kurtmuelleri*	Greece	Hofman et al. 2016, Mitochondrial DNA 27: 3434-3437
-	*P. kurtmuelleri*	Albania, Petrele	this study
-	*P. lessonae*	Europe	Dufresnes et al. 2017, Invasion Biol..
DQ902145	*P. perezi*	Spain	Busack and Lawson 2008, Biol J Linn Soc Lond 94: 445-461
-	*P. perezi*	Spain, Ronda	this study
-	*P. perezi*	Spain, Gran Canaria	this study
-	*P. perezi*	Spain, Trujilo	this study
-	*P. perezi*	Spain	this study
JN627421	*P. ridibundus*	Poland, Popowo	Hofman et al. 2012, Gene 491: 71-80
JN627423	*P. ridibundus*	Poland, Rafa	Hofman et al. 2012, Gene 491: 71-80
-	*P. ridibundus*	Switzerland, Graubünden	Dufresnes et al. 2017, Invasion Biol..
KP814012	*P. shqipericus*	Montenegro	Hofman et al. 2016, Mitochondrial DNA 27: 3434-3437

Supplementary Table 2: Mitochondrial (*cyt-b*) and nuclear (microsatellites) diversity for each taxon identified in the study area. n: sample sizes; H_d: haplotype diversity; π: nucleotide diversity; N_a: number of alleles; H_o: observed heterozygosity.

	MtDNA			Nuclear		
	n	H_d	π	n	N_a	H_o
P. perezi	27	0.721	0.00164	24	1.9	0.24
P. ridibundus	42	0.465	0.00053	58	5.4	0.54
P. kurtmuelleri	19	0.515	0.00106	36	7.5	0.65

Supplementary Table 3: Genetic distance (pairwise F_{st}) between populations with enough sample size ($n \geq 6$). Colors indicate the dominant species in each locality (green: *P. ridinbundus*; yellow: *P. kurtmuelleri*; red: *P. perezi*).

	1	2	4-5	8-9	10	11	12	17-18	23	24	26	27	28
1	-												
2	0.016	-											
4-5	0.000	0.024	-										
8-9	0.031	0.086	0.038	-									
10	0.166	0.216	0.158	0.050	-								
11	0.040	0.107	0.076	0.000	0.059	-							
12	0.000	0.010	0.012	0.023	0.028	0.000	-						
17-18	0.182	0.256	0.231	0.201	0.220	0.172	0.184	-					
23	0.306	0.372	0.351	0.331	0.338	0.334	0.317	0.021	-				
24	0.188	0.256	0.247	0.251	0.270	0.267	0.227	0.110	0.216	-			
26	0.198	0.287	0.258	0.241	0.284	0.261	0.274	0.004	0.121	0.178	-		
27	0.419	0.496	0.475	0.534	0.614	0.610	0.493	0.520	0.496	0.334	0.453	-	
28	0.417	0.492	0.472	0.520	0.613	0.591	0.476	0.485	0.424	0.269	0.403	0.110	-

Supplementary Figure 1: Phylogenetic analyses of a portion of *cyt-b* (514bp aligned), enabling comparisons with sequences covering the ranges and diversity of Eastern-Mediterranean marsh frogs (available on Genbank). Genbank accessions and origin of individuals are indicated. Haplotypes identified in this study (based on 975bp, see Figure 1 in the main text) are shown in color (bold: study area). Numbers show branch support (based on 1 000 bootstrap replicates) for major nodes.

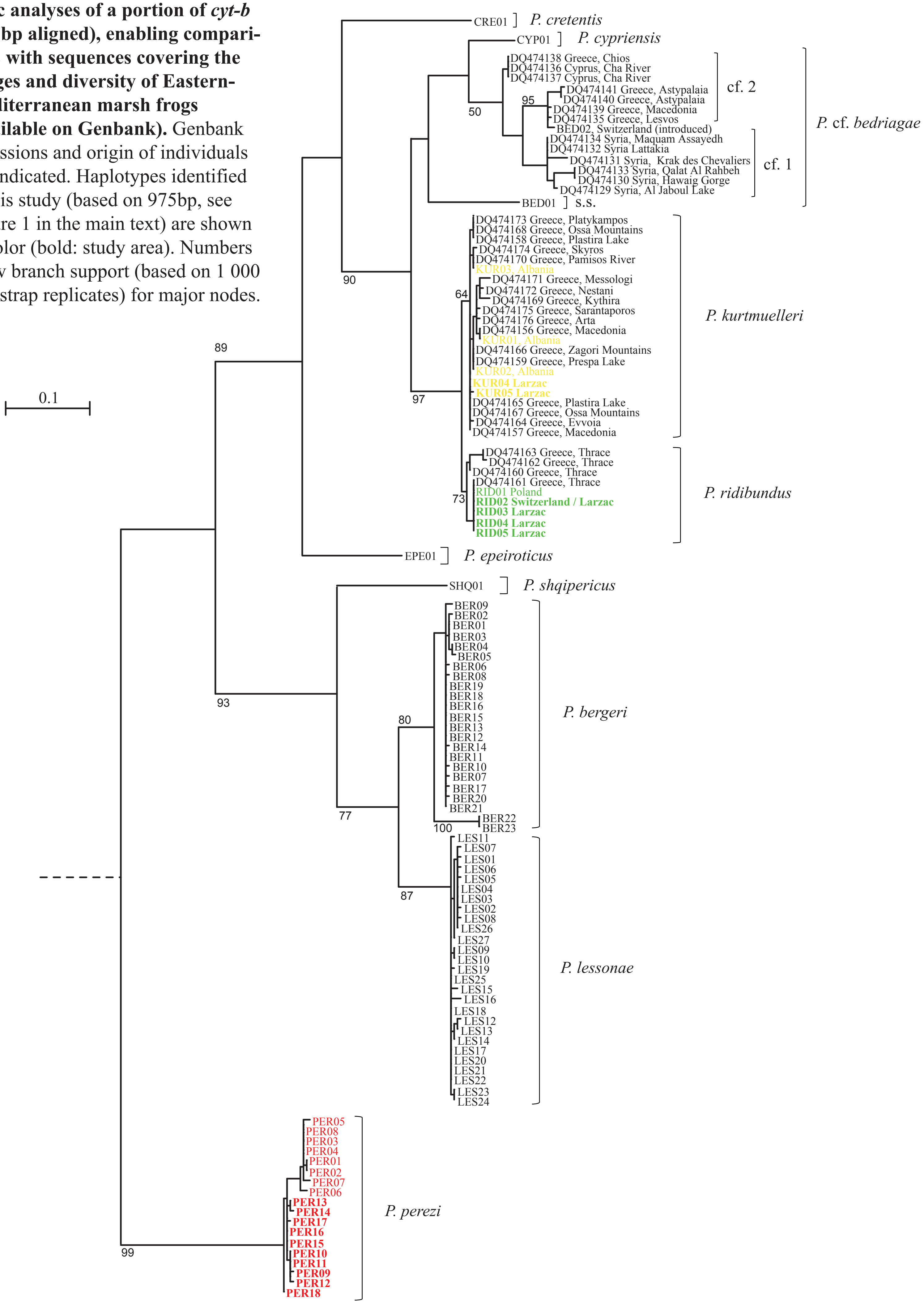

Supplementary Figure 2: Average log-likelihood Pr(X | K) and ΔK statistics of the STRUCTURE analyses. Barplots provide individuals assignments for fhe recommended Ks.

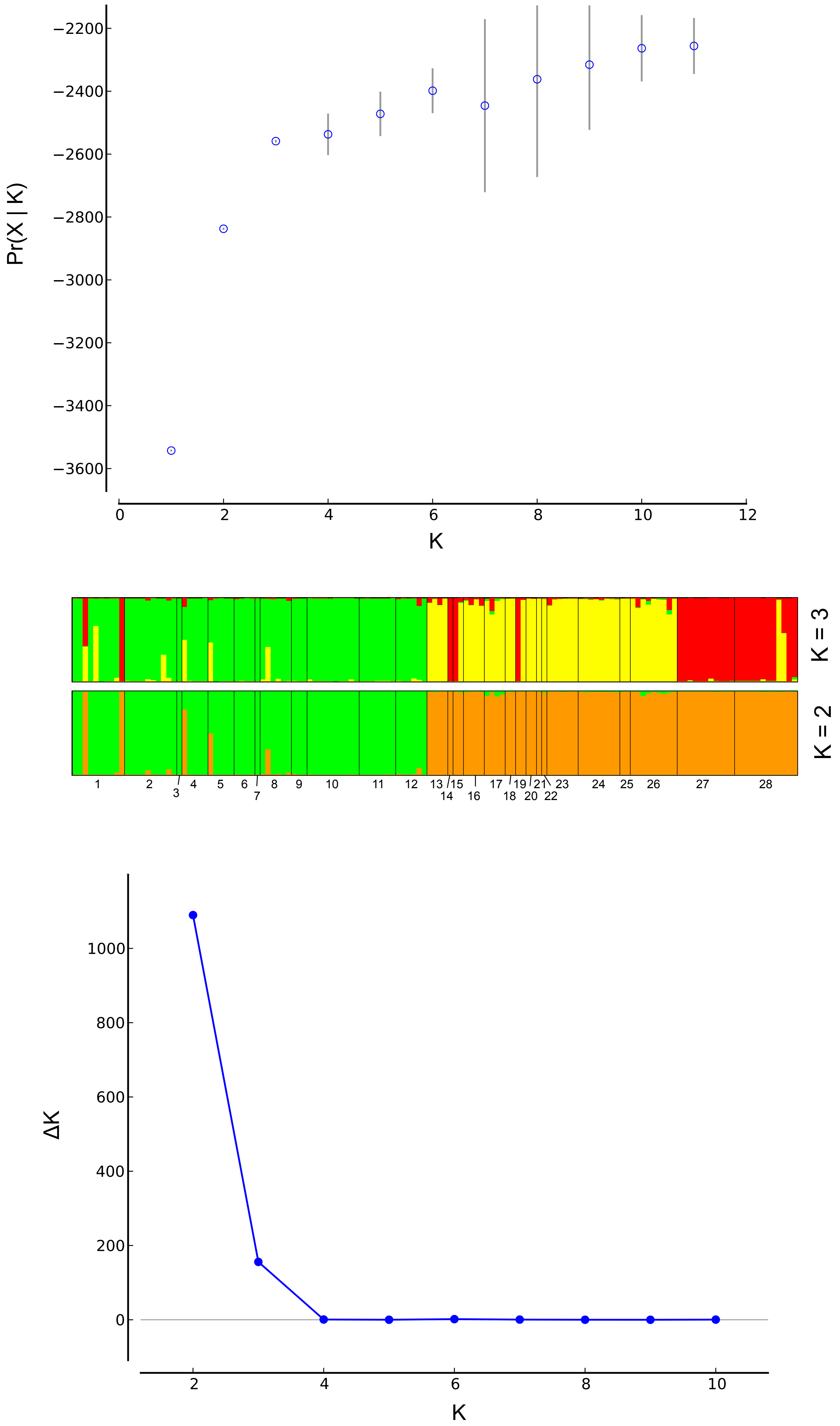

Supplementary Figure 3: Morphometric analyses. (A) PCA on morphometrics and (B) species comparisons. Significance of Tuckey's HSD tests are given: NS: non-significant, * p-value < 0.05. Crosses show the two *P. kurtmuelleri* × *P. perezi* hybrids, that may represent a new klepton.

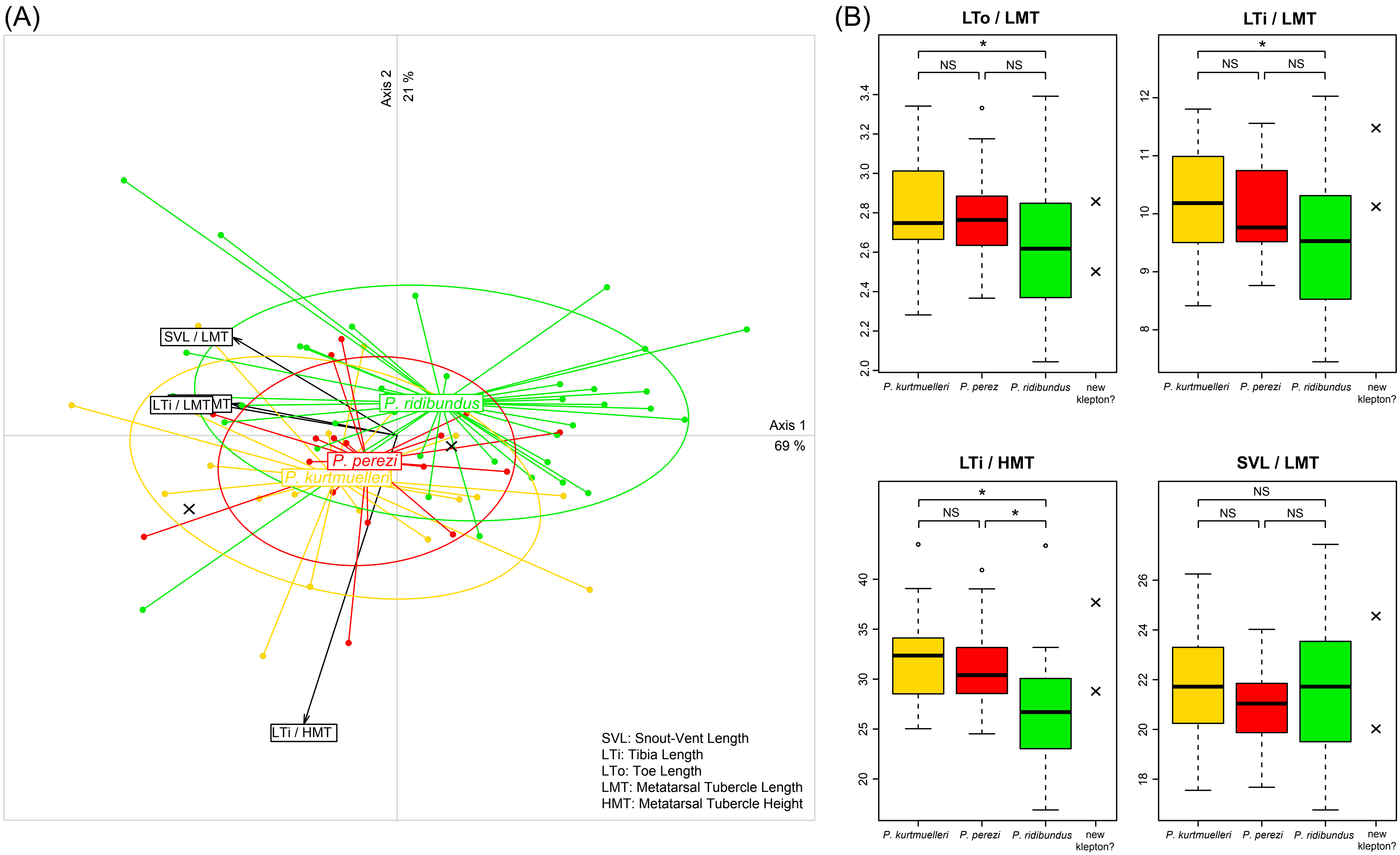

Supplement: Supplementary file 1 — Supplementary Information [file 41598_2017_6655_MOESM1_ESM.pdf]
